# A Case of Hemorrhagic Necrosis of Ectopic Liver Tissue within the Gallbladder Wall

**DOI:** 10.1155/2011/389381

**Published:** 2011-08-11

**Authors:** Sapna Nagar, Alan Koffron, Vandad Raofi

**Affiliations:** Department of Surgery, William Beaumont Hospital, Royal Oak, MI 48073, USA

## Abstract

Ectopic liver tissue is a rare clinical entity that is mostly asymptomatic and found incidentally. In certain situations, however, patients may present with symptoms of abdominal pain secondary to torsion, compression, obstruction of adjacent organs, or rupture secondary to malignant transformation. Herein, we report a case of a 25-year-old female that presented with acute onset of epigastric pain found to have ectopic liver tissue near the gallbladder complicated by acute hemorrhage necessitating operative intervention in the way of laparoscopic excision and cholecystectomy. The patient's postoperative course was uneventful. Gross pathology demonstrated a 1.2 × 2.8 × 4.5 cm firm purple ovoid structure that histologically revealed extensive hemorrhagic necrosis of benign ectopic liver tissue.

## 1. Introduction

Ectopic liver tissue is a rare entity that involves the presence of hepatic tissue in a number of sites outside of the native liver such as the gallbladder, hepatic ligaments, omentum, retroperitoneum, and thorax [1–5]. In most situations, they are asymptomatic and found incidentally. Rarely, they may present with symptoms of abdominal pain secondary to torsion, compression, or obstruction of adjacent organs, and rupture secondary to malignant transformation [1, 6, 7]. Herein, we report a case of ectopic liver tissue complicated by acute hemorrhage without any evidence of underlying malignancy.

## 2. Case Report

**Figure 1:**
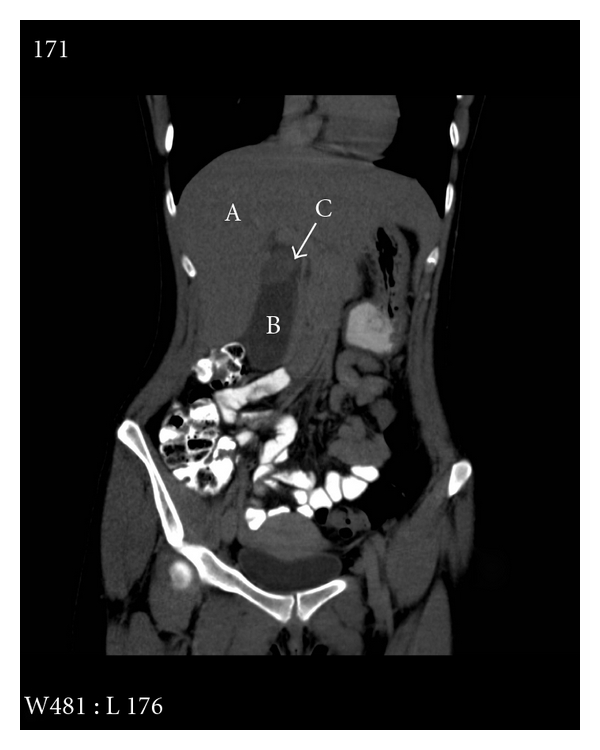
CT scan of Abdomen and Pelvis with soft tissue mass in proximity to the posterior wall of the gallbladder. A: liver, B: body of Gallbladder, and C: soft tissue mass.

An otherwise healthy 25-year-old female presented to our emergency department with complaints of acute onset of epigastric pain and nausea with bilious emesis. Workup in the way of liver function tests and abdominal ultrasound did not demonstrate evidence suggesting biliary or gallbladder pathology, and as such she was discharged home. She represented within hours of discharge due to worsening symptoms. She therefore underwent evaluation with a computed tomography that demonstrated a soft tissue mass located posterior to the gallbladder (Figure 1). The patient then underwent further evaluation with magnetic resonance cholangiopancreatography. Again, a complex cystic structure of indeterminate etiology was observed at the porta hepatis without evidence of communication with either the biliary or vascular structures. At the time, the differential diagnosis included a ruptured hepatic adenoma, biliary cystic lesion, or enteric cyst. 

**Figure 2:**
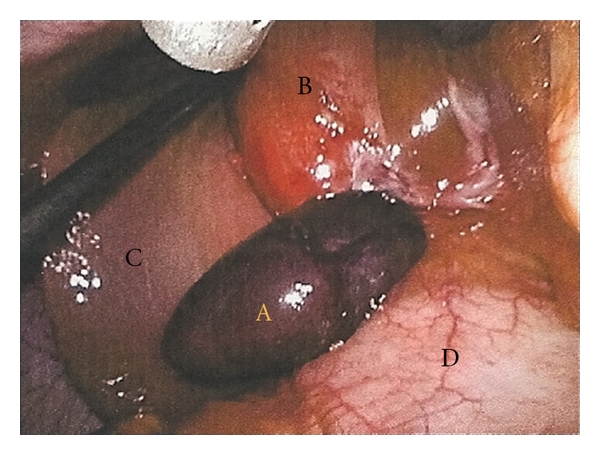
Hemorrhagic ectopic liver tissue at the time of laparoscopy. A: hemorrhagic ectopic liver, B: infundibulum of the gallbladder, C: right lobe of liver, and D: duodenum.

Secondary to these findings and her persistent symptoms the patient was taken to the operating room. At the time of laparoscopy a hemorrhagic pedunculated mass was identified attached to the infundibulum of the gallbladder once the fundus was retracted in a cephalad direction (Figure 2). It was easily dissected off the hilar structures by blunt dissection and then resected by ligating a small pedicle of tissue connecting this mass to the gallbladder infundibulum near the cystic duct by using a harmonic scalpel. There was also a considerable amount of inflammation to the infundibulum of the gallbladder in proximity to this hemorrhagic mass with the remainder appearing normal. Therefore, a cholecystectomy was performed as well. We believe the cholecystitis was secondary to inflammation from the hemorrhagic mass. As mentioned previously, her initial ultrasound did not show any evidence of cholecystitis. Even though one could expect this inflammation to resolve once the hemorrhagic mass was removed, we decided to perform a cholecystectomy because there was considerable dissection within the region of the cystic duct and the concern of potential ischemia and stricture did exist.

The patient's postoperative course was uneventful and her symptoms resolved immediately. She was discharged home on the first postoperative day and was asymptomatic when seen in follow-up.

**Figure 3:**
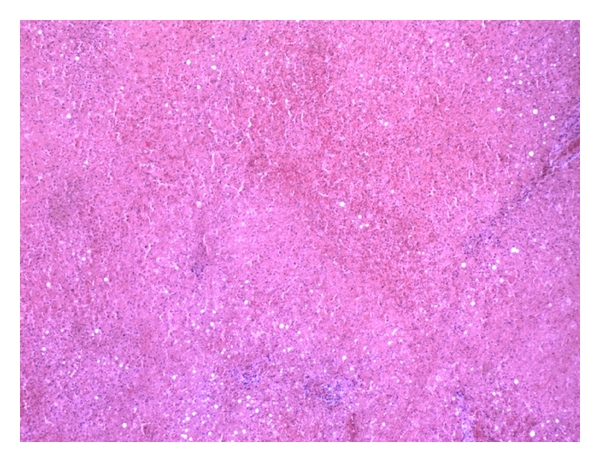
Histology of hilar mass revealing hemorrhagic necrosis in the background of normal liver parenchyma.

Gross pathology of the hilar mass revealed a 1.2 × 2.8 × 4.5 cm firm purple ovoid structure. Histology revealed extensive hemorrhagic necrosis in the background of normal liver parenchyma. The presence of hepatic lobules with central veins and organized portal structures ruled out adenoma and focal nodular hyperplasia (Figure 3). Therefore, the final diagnosis was that of a hemorrhagic benign ectopic liver tissue. Histologic evaluation of the gallbladder demonstrated localized cholecystitis in the area adjacent to the mass.

## 3. Discussion

Anomalous liver anatomy is classified as either accessory liver lobes where additional hepatic parenchyma is present outside of the native liver but is still connected to it or ectopic liver tissue where normal hepatic parenchyma is found outside of the liver with no hepatic connection [6]. The incidence of ectopic liver tissue has been reported to be anywhere from 0.24%–0.47% as diagnosed at the time of laparotomy or laparoscopy [1, 8]. In most situations, ectopic liver tissue is found on or near the gallbladder [2, 6]. A summary the cases presented in the literature including patient demographics, clinical presentation, location of ectopic liver tissue, and outcomes is presented in Table 1. 

Though there have been reports of ruptured ectopic liver tissue in the setting of malignant degeneration into hepatocellular carcinoma (HCC), this is the first case to our knowledge where benign hepatic tissue presented with symptomatic spontaneous hemorrhage [7]. 

There has been evidence to suggest that ectopic liver has a propensity to develop into malignancy. Theoretically, they may be susceptible to transformation to HCC secondary to their incomplete vascular and ductal systems that are incapable of acting in a metabolically appropriate manner. This altered physiologic response to normal stimuli, may facilitate their hepatocarcinogenesis. This theory may be further supported by the fact that the majority of cases where HCC is reported within ectopic liver tissue occur when there is no evidence of underlying cirrhosis or chronic inflammation of the native liver [2, 4, 9]. 

In summary, ectopic liver tissue can become symptomatic secondary to mass effect, spontaneous rupture, or malignant degeneration. Given these findings, we recommend resection of ectopic liver tissue when encountered. Unfortunately, the literature on this subject is limited and therefore it is difficult to draw any conclusions as to risk factors for development of complications such as location of tissue, use of oral contraceptives, and size of the lesion. With increased awareness of this topic and better imaging techniques, additional information may allow for recognition of risk factors and recommendations for surgical intervention.

## Figures and Tables

**Table 1:** Review of literature demonstrating clinical presentation and outcomes of patients with ectopic liver tissue.

Source	Age	Location	Presentation	AFP	Liver	Outcome
Watanabe et al. [1]	71 y.o. female 54 y.o. male 39 y.o. female 56 y.o. male 64 y.o female 37 y.o. male	Gallbladder Left lobe liver Retroperitoneum Gallbladder Gallbladder Gallbladder	Epigastric pain Malaise Abdominal Distension Malaise Epigastric pain incidental	Normal NA Abnormal Normal NA NA	Cirrhosis Cirrhosis Cirrhosis Cirrhosis Cirrhosis Cystic liver lesions	NA NA NA NA NA NA

Seo et al. [2]	59 y.o. male	Gastrorenal ligament	Incidental	Normal	NA	Discharged

Mehta et al. [3]	54 y.o. female	Right middle/lower lobe of lung	Post orthotopic heart transplant	NA	NA	Discharged

Kubota et al. [4]	56 y.o. male	Pancreas	DM/pancreatic tumor	NA	NA	Discharged

Kamath et al. [5]	37 y.o. female	Left lobe liver through diaphragmatic hernia	Abdominal pain	NA	NA	Discharged

Triantafyllidis et al. [6]	56 y.o. female	Gallbladder	Biliary colic	NA	NA	Discharged

Kuo et al. [7]	64 y.o. female	Triangular ligament lesser curve stomach	Acute onset pain internal bleeding	Abnormal	HCC	Expired

Arakawa et al. [9]	64 y.o. male 48 y.o. male	Left lobe liver Gallbladder	Incidental Autopsy	Abnormal NA	HCC Cirrhosis	Expired Expired

Hamadani et al.	49 y.o. male	Gallbladder	Incidental	NA	Cirrhosis	Discharged
